# Effects of caffeinated beverage ingestion on salivary antimicrobial proteins responses to acute exercise in the heat

**DOI:** 10.3389/fnut.2022.973003

**Published:** 2022-11-15

**Authors:** Lin Cheng, Hongli Wang, Yanbai Han

**Affiliations:** College of Physical Education and Health, Guangxi Normal University, Guilin, China

**Keywords:** caffeine, salivary antimicrobial proteins, acute exercise, heat, salivary α-amylase, salivary lactoferrin

## Abstract

Caffeine is commonly used by athletes as an energy supplement, but studies on its effects on salivary antimicrobial proteins (sAMPs) in humans during exercise are rare with ambiguous findings. It is also still controversial whether hot environments affect sAMPs. Using a double-blind, randomized crossover design, we examined 12 endurance-trained male collegiate athletes who completed the following two experiments: a caffeine experiment (CAF) and a placebo experiment (PLA). The participants acutely consumed caffeine-containing (6 mg/kg body weight) sports drink (3 ml/kg body weight) or an equivalent amount of placebo sports drink and subsequently performed cycling exercise for 40 min in the heat (33 ± 0.24°C, 64 ± 2.50% relative humidity) at 50% of maximum output power, maintaining a pedal frequency of 60 rpm. Saliva was collected at 60 min pre-exercise (T_–60_), the start of exercise (T_0_), 20 min of exercise (T_20_), and the end of the exercise (T_40_), and salivary α-amylase (sAA) and lactoferrin (sLac) were tested. The rating of perceived exertion (RPE) was measured at T_0_–T_40_, while core body temperature (T_*re*_) and heart rate (HR) were monitored continuously. T_*re*_, HR, and RPE increased with time during the exercise (*p* < 0.01), with no difference in T_*re*_ and HR between the CAF and PLA (*p* > 0.05), but RPE was higher in the PLA than in the CAF (*p* < 0.05). sLac concentrations were significantly higher at T_20_ and T_40_ than at T_–60_ (*p* < 0.01) and higher at T_40_ than at T_0_ and T_20_ (*p* < 0.01), with no difference between the CAF and PLA (*p* > 0.05). Compared with T_–60_, sAA activity was significantly increased at T_0_, T_20_, and T_40_ (*p* < 0.01). sAA activity was significantly higher at T_40_ than at T_0_ and T_20_ (*p* < 0.01), at T_20_ than at T_0_ (*p* < 0.05), and in the CAF than in the PLA (*p* < 0.01). Heat stress caused by acute exercise in hot environments did not impair the sAMPs parameters of the participants. Instead, the participants showed transient increase in sAA activity and unchanged sLac concentrations. Caffeine may increase salivary markers related to immune response during exercise.

## Introduction

Exercise plays an important role in immunity (1). Studies have confirmed that regular medium-intensity exercise can enhance immune function, and prolonged high-intensity training can cause immunosuppression (2). Athletes in various sports disciplines frequently report symptoms associated with upper respiratory tract infections (URTI) during extensive training and competition, which could lead to fever and dehydration, reduced energy and protein stores, decreased muscle strength, and reduced exercise performance. Mucosal immunity serves as the first line of defense against URTI (3). The expression of salivary antimicrobial proteins (sAMPs), which is a prime factor in determining mucosal immunity (4), can be regulated by exercise (5–7). sAMPs play both antimicrobial and immunomodulatory roles. Regarding their antimicrobial roles, they act directly on microorganisms by killing them, inhibiting their growth and activity, or preventing them from initiating inflammatory responses. Regarding their immunomodulatory roles, sAMPs participate in the immune response by recruiting cells, inducing cytokines, and helping in tissue repair (8).

sAMPs can be used as parameters to assess the effects of exercise on mucosal immunity (9). The upper respiratory tract is protected by sAMPs, which includes salivary lactoferrin (sLac), salivary α-amylase (sAA), lysozyme, etc., (10). sLac, one of the most abundant sAMPs, is an iron-binding glycoprotein secreted by the plasma glandular vesicle cells of the salivary glands (11). It performs its antibacterial function by sequestering iron away from bacteria to prevent their growth (12). sLac inhibits the secretion of tumor necrosis factor (TNF)-α, interleukin (IL)-1β, IL-6, and IL-8 from monocytes and is a marker of innate mucosal immunity (13). sAA is also one of the most abundant proteins in saliva, accounting for 40–50% of proteins in saliva. It is an innate mucosal immune marker with antimicrobial properties of inhibiting the adhesion and growth of bacteria (14). In addition, sAA is often used as a biomarker of physical stress and sympathetic nervous system activity (15). Previous studies have examined sLac and sAA and their potential relationship with respiratory symptoms in terms of different exercise duration, intensities, and types. A study showed no change in the sLac concentrations of 47 non-elite marathoners after they completed marathon (16). Another study showed that 14 endurance athletes had significantly higher sLac concentrations after completing a 50-km ultramarathon race compared with the concentrations before the race (17). In addition, a study that recruited eight basketball players showed decrease in sLac concentrations during training and competition (18); Wunsch et al. (19) reported in their study that 84 male participants aged 18–30 years showed increased sAA activity after moderate-to-high ergometer cycling. Walsh et al. (20) reported that eight trained men experienced a decrease in sAA activity immediately after cycling. To date, studies on the effects of exercise on sLac and sAA are relatively scarce, with numerous associated controversies that require further research.

Many major sporting events, such as the Summer Olympics and the FIFA World Cup, are held in hot environments. It has been shown that increased sympathetic excitation during exercise in a hot environment (>30^°^C) can cause dehydration, decreasing salivary flow rate, which subsequently decreases mucosal surface sAMPs concentration and increases the risk of URTI (21–23). A study reported that 10 healthy men showed increased sLac concentrations after running on a treadmill in a hot and humid environment (40°C and 50% relative humidity) at a 95% ventilatory threshold (24). In addition, sAA activity increased in eight males after running at 60% VO_2m*ax*_ for 2 h for 7 consecutive days at 34^°^C and 32% relative humidity (25). Similarly, there are fewer studies on the effects of exercise in hot environments on sAMPs response, and the conclusions on its effects on mucosal immune function are still controversial. In sports research, it is important to assess how the incidence of URTI can be reduced to avoid it affecting exercise performance during training or competition in hot environments.

Caffeine (1,3,7-trimethylxanthine) is present in many plants and is often added to various foods and beverages as an energy aid, increasingly becoming one of the most consumed psychoactive substances (26). After World Anti-Doping Agency removed caffeine from the list of prohibited substances, it has commonly been used by athletes to enhance exercise capacity and performance. Studies have shown that consumption of 3–6 mg/kg body weight of caffeine can improve exercise performance (27, 28). In addition to the effect caffeine has on the central nervous system, it also has effects on the digestive, respiratory, urinary tract, and other systems (26). Such effects are its correlation with a lower risk of Alzheimer’s disease (29, 30) and Parkinson’s disease (31), its ability to control diabetes (32), its antioxidant properties (33), etc. Caffeine, like other members of the methylxanthine family, is largely anti-inflammatory and modulates innate and adaptive immune responses (34). Few controversial studies have explored the effects of caffeine on mucosal immunity in athletes. One study showed that green tea (containing 6 mg/kg body weight of caffeine) consumption by taekwondo athletes after training sessions increased sAA activity (35), whereas Klein et al. (36) found that caffeine did not alter sAA activity. Salivary secretion is regulated by the autonomic nervous system, and the salivary glands are innervated by branches of the parasympathetic and sympathetic nervous systems. Caffeine intake is associated with enhanced sympathetic nervous system activity and increased plasma adrenaline (16, 20). Therefore, it can be hypothesized that it can cause salivary response to exercise. The effect of caffeine supplementation on sAA and sLac in humans during exercise in a hot environment is still unclear and needs further study.

Therefore, the present study focused on investigating the effects of caffeine intake (6 mg/kg) and hot environments on sAA and sLac after moderate intensity exercise in male endurance-trained college athletes. We hypothesized that exercise in a hot environment may decrease sAMPs concentrations, while caffeine intake may enhance the expression of human mucosal immune markers.

## Materials and methods

### Participants

Twelve healthy college students with long-term participation in regular endurance training (mean ± standard deviation; age: 21.17 ± 1.07 y, height: 180.42 ± 5.22 cm, weight: 72.03 ± 6.55 kg, VO_2m*ax*_: 50.19 ± 9.54 ml/kg/min, and average training years 2.89 ± 1.14 y) were recruited. Inclusion criteria: (a) no heat acclimation; (b) no history of cardiovascular, metabolic, or respiratory disease; (c) no supplements or medications that affect thermoregulation; (d) aerobic training of not less than 4 times per week. Participants signed an informed consent form after understanding the study content, procedure, and potential risks in detail. This study strictly complies with the Declaration of Helsinki and was approved by the Ethics Committee of Guangxi Normal University. Participants were required to maintain a healthy lifestyle throughout the experimental period and to avoid strenuous exercise, alcohol consumption, and caffeine intake for 24 h before the trials. They were also instructed to maintain and record their patterns of physical activity and dietary habits prior to the familiarization experiment and to repeat this practice in subsequent experiments. Diet was prohibited for 2 h prior to the trial, and three meals were standardized (carbohydrate, 50–65%; protein, 10–15%; and fat, 20–30% in one meal).

### Preliminary testing

Participants first visited the laboratory where air displacement plethysmography (BOD POD) (Life Measurement Instruments, Concord, Calif.) was used to measure body composition (percentage of body fat). The participants wore a bathing suit and bathing cap, and they were instructed to remove all jewelry. All participants were relaxed and comfortable in the BOD POD chamber and were asked to breathe normally during the 5 min test. Subsequently, we performed an incremental exercise test to volitional exhaustion using a cycle ergometer (MONARK 839E, Varberg, Sweden) to determine the maximum power. Prior to the measurements, participants adjusted and recorded the height of the bike seat for two subsequent trials and wore a heart rate (HR) band. They started cycling at 0 kp and increased the load by 0.25 kp every 15 s until volitional fatigue, maintaining a speed of 60 rpm. After a 10 min break, participants were familiarized with the processes of caffeine or placebo administration, exercise protocol, and saliva collection to ensure that they were accustomed to the procedures used in the two formal trials.

### Experimental procedures

**FIGURE 1:**
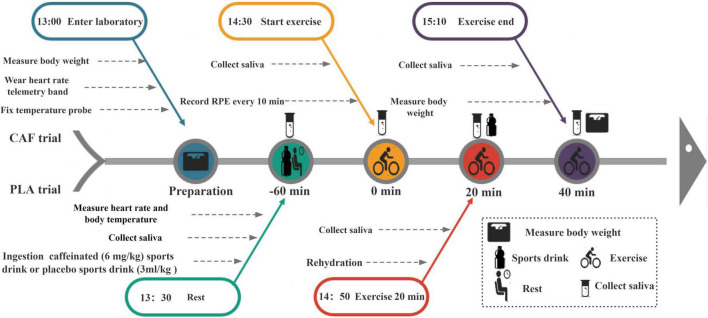
Experimental procedure chart.

A double-blind, randomized crossover experimental design was used in this study, which included 3 sessions. Seven to ten days after the familiarization test, the participants completed two experiments [a caffeine experiment (CAF) and placebo experiment (PLA)], which were separated by at least a 1-week interval. All experiments were performed between 13:00 and 15:10 to eliminate the effect of circadian rhythm changes. For both trials, the participants entered the laboratory after a 2 h fast. Upon arrival, the participants first emptied their bladder, and their nude body mass was measured (AD6205B, A&D Co., Ltd., Tokyo, Japan). Next, the participants self-inserted a rectal thermometer into the anal sphincter at a distance of 10 cm and fixed a HR telemetry band (Polar Beat, Kempele, Finland) around the chest. Saliva baseline samples were collected while seated on a chair, and then participants acutely ingested a sports drink (3 ml/kg body weight) with 6 mg/kg caffeine (200 mg/capsule, Prolite, USA) or an equivalent placebo sports drink (MI zone beverage, Danone, China). Peak plasma caffeine concentrations occur at approximately 30–60 min (37); therefore, according to previous studies, participants remained seated from 13:30 for 60 min (38). At 14:28, a second saliva sample was collected. Immediately at 14:30, participants began cycling at 50% maximum power for 40 min in a climate chamber (33 ± 0.24°C and 64 ± 2.50% relative humidity), maintaining the speed at 60 rpm. The mean workload of participants was 1.92 ± 0.43 kp. At 14:50 (20 min of exercise), a third saliva collection was performed. To avoid the effect of dehydration on saliva composition, the participants were supplemented with a sports drink (3 ml/kg body weight) after the saliva was collected. At 15:10 (end of the exercise), the fourth saliva sample was collected for testing. Finally, the participants dried their body sweat and measured their post-exercise weight. The experimental procedure is shown in Figure 1.

### Measurements

#### Physiological indices measurement

A wet-bulb globe thermometer (WBGT-213A/213B) was used to monitor ambient temperature and relative humidity during each trial. Before and after each trial, participants (in shorts) were weighed (XK3150, EXCELL, Shanghai, China). The rectal probe thermometer (YSI401AC, USA) was attached to a temperature monitor to measure the participants’ core body temperature (T_*re*_), which was logged at 1 min intervals by a data logger (YSI Precision*™*, USA). HR was recorded every 1 min throughout the test period. Rating of perceived exertion (RPE) was recorded every 10 min from the beginning to the end of the exercise using a Borg scale (39). The total sweat volume was calculated using the following formula: body mass before the trial + the amount of the ingested drink – body mass after the exercise.

#### Saliva collection and analysis

Participants’ mouths were examined to ensure there were no bleeding wounds; during sample collection, they were seated and instructed to rinse their mouths twice with water. Saliva samples were collected at the following four points: 60 min before the exercise (T_–60_), at the start of exercise (T_0_), at 20 min of exercise (T_20_), and immediately after the exercise (T_40_). For sample collection, participants chewed the swab at a rate of once per second for 1 min. The saliva produced was collected into pre-weighed test tubes (Salivettes^®^; Sarstedt, Nümbrecht, Germany) using a cotton swab. The cotton swab was placed back into the saliva collection tube, and the tubed was centrifuged (TDL80-2B, Shanghai, China) at 3,800 rpm for 20 min. Subsequently, saliva samples were weighed for saliva volume estimation and stored at −80°C. Saliva flow rate was calculated by dividing the volume collected by the time duration for collection. Analysis of sLac concentration was performed using an enzyme-linked immunosorbent assay according to the manufacturer’s instructions (human lactoferrin, Nanjing Jiancheng Bioengineering Institute, Nanjing, China). sAA activity was determined using amylopectin-iodine colorimetric assay (α-Amylase Assay Kit, Nanjing Jian Cheng Bioengineering Institute, Nanjing, China). The intra-assay coefficient of variation for sLac and sAA were 7.8 and 4.2%, respectively. Two samples were taken from each participant, and the concentration for all the samples were measured on the same plate.

### Statistical analysis

G*Power 3.1.9.7, with an effect size of 0.40, an alpha value of 0.05, and a power of 0.85, was used to estimate the sample size. Based on the calculation, the minimum sample size necessary to satisfy the test requirements was 12. All data were statistically analyzed using SPSS 25.0 software (Chicago, IL, USA) and expressed as mean ± standard deviation. The Shapiro-Wilk test and Levene tests showed that the data were normally distributed and the variance was homogeneous. Body weight and sweat loss were compared using paired *t-test*. T_*re*_, HR, RPE, sAA, and sLac were analyzed using (condition × time) repeated measures ANOVA, corrected by the Greenhouse–Geisser method when the sphericity test was violated. If significant differences were found, the Bonferroni *post hoc* test was used. Significant differences were considered at *p* < 0.05.

## Results

### Salivary responses

**FIGURE 2:**
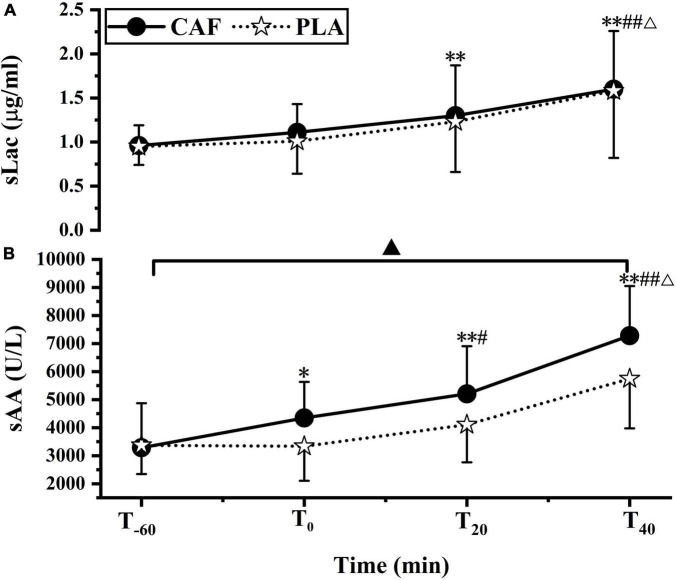
**(A)** Salivary lactoferrin concentration, **(B)** salivary α-amylase activity responses to CAF and PLA. Values are means ± standard deviation (*n* = 12). *p < 0.05, ^**^p < 0.01 vs. T_–60_; ^#^*p* < 0.05; ^##^*p* < 0.01 vs. T_0_; ^Δ^*p* < 0.01 vs. T_20_, ^▲^*p* < 0.05 vs. PLA.

sLac concentration increased significantly at T_20_ (CAF: 1.30 ± 0.57, PLA: 1.23 ± 0.57) (*p* < 0.01) and T_40_ (CAF: 1.60 ± 0.66, PLA: 1.58 ± 0.76) (*p* < 0.01) compared with that at T_–60_ (CAF: 0.96 ± 0.23, PLA: 0.95 ± 0.21, *p* = 0.871). The sLac concentration at T_40_ was higher than that at T_0_ (CAF: 1.11 ± 0.32, PLA: 1.01 ± 0.37) (*p* < 0.01) and T_20_ (*p* < 0.01). No differences were observed in the change in sLac concentration between the CAF and PLA (*p* = 0.747) (Figure 2A).

Compared to T_–60_ (CAF: 3285 ± 1588, PLA: 3367 ± 1020, *p* = 0.882), sAA activity was significantly increased at T_0_ (CAF: 4347 ± 1284, PLA: 3338 ± 1231) (*p* < 0.05), T_20_ (CAF: 5203 ± 1702, PLA: 4107 ± 1345) (*p* < 0.01) and T_40_ (CAF: 7281 ± 1769, PLA: 5746 ± 1769) (*p* < 0.01). sAA activity was higher at T_40_ than at T_0_ and T_20_ (*p* < 0.01) and was significantly higher at T_20_ than at T_0_ (*p* < 0.05). sAA activity in the CAF was higher than that in the PLA (*p* < 0.05) (Figure 2B).

**FIGURE 3:**
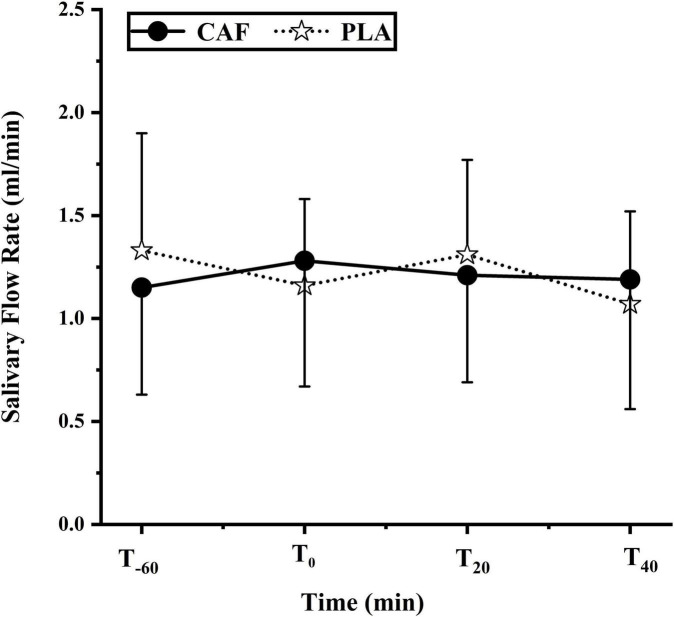
Saliva flow rate during the two trials of the participants. Data are mean ± standard deviation (*n* = 12).

Compared with T_–60_ (CAF:1.15 ± 0.55, PLA:1.33 ± 0.60, *p* = 0.454), there was no significant change in salivary flow rate at T_0_ (CAF:1.28 ± 0.64,PLA:1.16 ± 0.44) (*p* = 0.733),T_20_ (CAF:1.20 ± 0.55,PLA:1.31 ± 0.48)(*p* = 0.824), T_40_ (CAF:1.19 ± 0.66,PLA:1.07 ± 0.47) (*p* = 0.162). Salivary flow rate did no differ between trials (*p* = 0.958) (Figure 3).

### Core temperature

**FIGURE 4:**
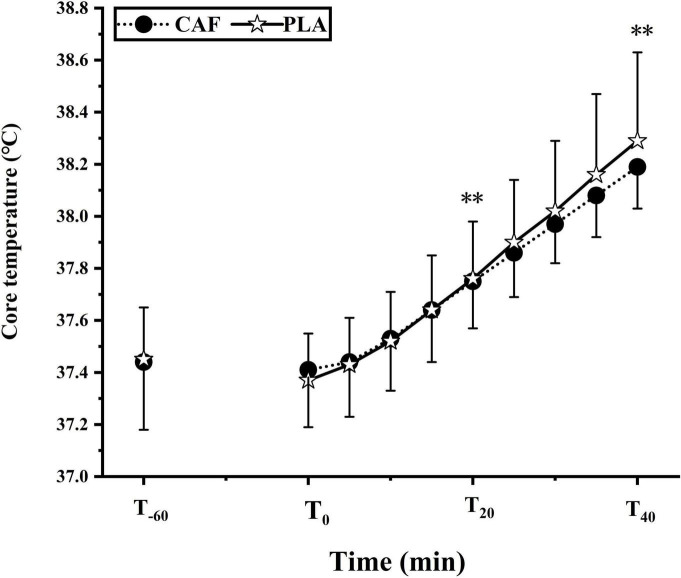
The change of core temperature in two trials. Data are mean ± standard deviation (*n* = 12). ^**^p < 0.01 vs. T_0_.

At T_0_, the T_*re*_ in the CAF and PLA was 37.37 ± 0.18°C and 37.41 ± 0.22°C (*p* = 0.448), respectively. T_*re*_ increased linearly (peaked at CAF: 38.19 ± 0.16°C vs. PLA: 38.29 ± 0.34°C) during the exercise (*p* < 0.01). T_*re*_ was unaffected by caffeine ingestion as it was similar between the CAF and PLA throughout the exercise (*p* = 0.913) (Figure 4).

### Sweat loss

In both trials, the participants lost weight after the exercise compared to their pre-exercise weight (*p* < 0.01). There was no significant difference in sweat volume between the two trials (*p* > 0.05), and the percentage weight loss in the CAF and PLA was < 1%, indicating that the participants were not dehydrated during the experiments (Table 1).

**TABLE 1:** Changes in sweat volume and weight loss of the participants during trials.

Conditions	Pre-exercise weight (kg)	Post-exercise weight (kg)	Sweat volume (kg)	Weight loss (%)
CAF	72.03 ± 6.84	71.63 ± 6.80**	0.40 ± 0.12	0.55 ± 0.18
PLA	72.13 ± 6.67	71.78 ± 6.63**	0.35 ± 0.12	0.48 ± 0.14

Data are mean ± standard deviation (*n* = 12). **p < 0.01 vs. pre-exercise weight.

### Heart rate and rating of perceived exertion

During the exercise, HR and RPE increased over time in both trials compared to the HR and RPE at T_0_ (*p* < 0.01). There was no significant difference in HR between the two trials (*p* = 0.583); however, RPE in the CAF was lower than in the PLA (*p* < 0.05) (Tables 2, 3).

**TABLE 2:** Heart rate responses over the entire trial (beats/min).

Measurements	Conditions	T_–60_	T_0_	T_20_	T_40_
HR	CAF	81.08 ± 14.06	76.17 ± 13.52	139.92 ± 13.22**	153.67 ± 15.14**^#^
	PLA	80.17 ± 14.75	83.25 ± 12.73	142.50 ± 14.86**	154.42 ± 15.71**^#^

Data are mean ± standard deviation (*n* = 12). **p < 0.01 vs. T_0_; ^#^*p* < 0.01 vs. T_20_.

**TABLE 3:** Changes in RPE during exercise in both groups.

Measurements	Conditions	T_0_	T_10_	T_20_	T_30_	T_40_
RPE	CAF	8.75 ± 1.60	9.42 ± 1.78	10.17 ± 1.95**^##^	11.00 ± 2.17**^##ΔΔ○○^	12.25 ± 2.86**^##ΔΔ○○▲^
	PLA	8.67 ± 2.10	10.58 ± 2.39**	11.75 ± 1.87**	13.42 ± 1.83**^##ΔΔ^	14.83 ± 2.79**^##ΔΔ▲▲^

Data are mean ± standard deviation (*n* = 12). **p < 0.01 vs. T_0_; ^##^*p* < 0.01 vs. T_10_; ^ΔΔ^*p* < 0.01 vs. T_20_; ^▲▲^*p* < 0.01 vs. T_30_; ^○^*p* < 0.05, ^○○^*p* < 0.01 vs. PLA.

## Discussion

This study showed that participants had increased T_*re*_ during the exercise in a hot environment (ΔT_*re*_ < 1°C; T_*re*_ > 38°C at the end of exercise) and that their sLac and sAA responses to exercise were similar to those reported in previous studies that were conducted in thermoneutral conditions (40). Furthermore, our results showed that supplementation with 6 mg/kg body weight of caffeine did not alter salivary flow rate, HR, or sLac concentration but increased sAA activity.

Saliva, compared with plasma and serum, can be collected quickly, frequently, and non-invasively for a wide range of applications in sports science (41). Studies have rarely investigated if changes in the sAMPs response (e.g., sAA and sLac) alter susceptibility to infection. In our experiment, the participants consumed caffeine (6 mg/kg) and subsequently exercised in a hot environment for 40 min, causing an increase in T_*re*_ of about 1°C (T_*re*_ > 38°C at the end of the exercise, Figure 4) and in sLac concentration in both trials, which is consistent with the findings of McKenna et al. (24). Another study also showed that sLac concentration increased in eight participants after 45 min of running on a treadmill at 75% VO_2m*ax*_ (42). The results suggested that exercise may activate neutrophils to release lactoferrin into saliva (43), which may have increased sLac concentration and further modulated mucosal surface inflammation. Meanwhile, moderate-intensity exercise may promote anti-inflammation by reducing toll-like receptors and increasing circulating T regulatory cells, in addition to reducing the release of pro-inflammatory cytokines in circulating cells, muscles, and adipose tissues (44). The effect of a hot environment on sLac concentration was consistent with the results of 11 men obtained after 45 min of running at 75% VO_2m*ax*_ under thermoneutral conditions (45). Our result showed that sLac concentration was less affected by exercise in a hot environment and may not impair mucosal immune function. Another study showed that sLac concentration was unchanged in 16 elite male taekwondo athletes for a 7-week training (46), while 10 female taekwondo (without rapid weight changes) decreased sLac concentration after the competition (47). In addition, Li et al. (48) found no difference in lactoferrin levels between 12 male adolescent volleyball players and a sedentary control group after high-intensity training. These studies have shown that different changes in sLac concentrations during exercise are associated with age and sex, environmental conditions, type of exercise, and exercise intensity. No difference in sLac concentrations was observed between the CAF and PLA. This result may be associated with changes in sympathetic activity and not with the hypothalamic-pituitary-adrenal axis activity (49). Caffeine consumption is associated with enhanced sympathetic nervous system activity and increased plasma adrenaline (50, 51), and its mechanism of action needs to be further studied due to the lack of relevant studies.

sAA activity is regulated by the sympathetic nervous system, with most of the proteins produced by the parotid glands (52). Antimicrobial proteins (sLac and sAA) act synergistically to protect the upper respiratory tract from pathogenic invasion, similar to the protective properties of IgA in saliva (53). The activity of sAA is related to the intensity and mode of exercise (54). Yasuda et al. (55) found that 11 male cyclists had increased sAA activity after exercising at 60% of peak oxygen uptake in a thermoneutral environment. Another study showed increased sAA activity in adolescent swimmers after swimming lessons (56). Gill et al. (53) also found that 23 ultra-endurance runners had increased sAA activity after a 230-km multi-stage ultramarathon in hot environmental conditions (32– 40°C). In the present trial, compared with T_–60_, sAA activity increased with time during the exercise, which is consistent with the above above-mentioned previous findings and shows that the sAA activity responses in a hot environment were the similar to those in a thermoneutral environment. In addition, 11 endurance-trained male athletes consumed 6 mg/kg body weight of caffeine or placebo 1 h prior to exercise, followed by a 90 min cycling at 70% VO_2m*ax*_, showed increased sAA activity at rest and during the exercise (57), which was consistent with the results of the present experimental study. An increase in sAA activity at rest and during exercise could indicate that salivary secretion is induced primarily by adrenergic stimulation. Caffeine is a potent stimulator of the sympathetic nervous system, increasing its activity by blocking adenosine receptors and releasing plasma catecholamines. Following adenosine binding to A2 receptors, adenosine cyclase activity is enhanced by inhibition of phosphodiesterase, thereby inducing an intracellular signaling cascade mediated by cyclic adenosine monophosphate (cAMP) upregulation. Thus, the extracellular binding of caffeine is amplified intracellularly by the second messenger cAMP. High concentrations of cAMP activate protein kinase A, which inhibits lipopolysaccharide-stimulated production of pro-inflammatory cytokines such as tumor necrosis factor (TNF-α) and interleukin (IL-12), thereby preventing the release of pro-inflammatory cytokines and promoting the production of anti-inflammatory cytokines (58). Adenylyl cyclase activity can also cause adrenergic stimulation causing high salivary organic content including elevated total protein levels, especially sAA activity. In addition, caffeine reduces TLR1 and TLR2 expressions and promotes anti-inflammation (58) and enhances mucosal immunity and control of inflammation.

Salivary composition and secretion are controlled by the autonomic nervous system (59), which in turn indirectly regulate the salivary flow rate (21). The salivary flow rate depends mainly on the type of activated autonomic receptors, which in turn affects saliva composition (9). A study have shown that dehydration affects level of sAMPs (22). Therefore, in this study rehydration was performed at 20 min of exercise to maintain normal hydration, and the sweat volume of the participants in this study less than 1%, indicating the absence of dehydration (Table 1). Staying euhydrated can reduce the risk of UTRI (60). The present study showed that RPE was significantly lower in the CAF than in the PLA. The present results were consistent with those of previous results that caffeine reduced RPE independent of ambient temperature (61, 62). Caffeine acts as an adenosine receptor antagonist that affects the central nervous system (63), leading to delayed fatigue and improved exercise performance (64). The effect of caffeine on human heart rate during exercise is still controversial. Stadheim et al. (65) found that caffeine enhances the activity of the sympathetic nervous system, causing an increase in HR. In contrast, other studies have shown that oral caffeine can lower HR during exercise (66, 67). Other studies have also shown that caffeine did not affect HR when the participants exercised at 33^°^C or 37^°^C (68, 69), which was consistent with our findings. Therefore, the effect of caffeine ingestion on HR during exercise needs to be further investigated under different ambient temperatures and with different forms of exercise.

## Limitations

There are limitations to this study. First, caffeine absorption varies individually and we did not examine plasma caffeine concentrations. Second, the increase in sAA activity caused by caffeine may be due to increased plasma levels of catecholamines, and we did not test blood adrenaline and norepinephrine concentrations. Third, there are various sAMPs, but only sLac and sAA were chosen for testing. In future studies, different doses of caffeine could be administered orally, and different intensities of exercise could be performed in different temperature environments to observe the responses of lysozyme, immunoglobulin A, immunoglobulin M, sLac, and sAA.

## Conclusion

The current findings suggest that ensuring euhydration during exercise in hot environments may not pose a greater threat to sAMPs parameters. In addition, participants supplemented with caffeine (6 mg/kg) showed no change in sLac concentration, but caused increase in sAA activity, and therefore ensuring adequate levels of sAMPs.

## Data availability statement

The original contributions presented in this study are included in the article/Supplementary material, further inquiries can be directed to the corresponding author.

## Ethics statement

The studies involving human participants were reviewed and approved by the College of Physical Education and Health, Guangxi Normal University. The patients/participants provided their written informed consent to participate in this study.

## Author contributions

YH and HW designed and carried out the experiments. LC analyzed the data and wrote the manuscript. All authors have read and agreed to the published version of the manuscript.
