# Supplementary material for: Effects of caffeinated beverage ingestion on salivary antimicrobial proteins responses to acute exercise in the heat
Source: Front Nutr. 2022 Nov 15;9:973003. doi: 10.3389/fnut.2022.973003 (PMC9705747; doi:10.3389/fnut.2022.973003)
Supplement: Supplementary file 1 [file Data_Sheet_1.ZIP › date/sAA.pdf]

rial number	Sample number	OD	dilution ratio	amylase activity （U/dl）
	standard	0.532		
1	A1(Zilun Zheng)	0.405	3000	57293.2331
2	A2(Zilun Zheng)	0.436	4500	64962.4060
3	A3(Zilun Zheng)	0.399	3000	60000.0000
4	A4(Zilun Zheng)	0.41	6000	110075.1880
5	a1(Zilun Zheng)	0.442	3000	40601.5038
6	a2(Zilun Zheng)	0.456	3000	34285.7143
7	a3(Zilun Zheng)	0.438	3000	42406.0150
8	a4(Zilun Zheng)	0.429	4500	69699.2481
9	B1(Qiang Bian)	0.457	3000	33834.5865
10	B2(Qiang Bian)	0.421	3000	50075.1880
11	B3(Qiang Bian)	0.408	3000	55939.8496
12	B4(Qiang Bian)	0.399	4500	90000.0000
13	b1(Qiang Bian)	0.429	3000	46466.1654
14	b2(Qiang Bian)	0.411	3000	54586.4662
15	b3(Qiang Bian)	0.444	3000	39699.2481
16	b4(Qiang Bian)	0.425	4500	72406.0150
17	C1(Bowen Xing)	0.378	1500	34736.8421
18	C2(Bowen Xing)	0.389	2400	51609.0226
19	C3(Bowen Xing)	0.455	3000	34736.8421
20	C4(Bowen Xing)	0.437	3000	42857.1429
21	c1(Bowen Xing)	0.468	3000	28872.1805
22	c2(Bowen Xing)	0.47	3000	27969.9248
23	c3(Bowen Xing)	0.445	3000	39248.1203
24	c4(Bowen Xing)	0.377	3000	69924.8120
25	D1(Guiming Yang)	0.479	3000	23909.7744
26	D2(Guiming Yang)	0.473	3000	26616.5414
27	D3(Guiming Yang)	0.409	3000	55488.7218
28	D4(Guiming Yang)	0.438	4500	63609.0226

29	d1(Guiming Yang)	0.482	3000	22556.3910
30	d2(Guiming Yang)	0.478	1500	12180.4511
31	d3(Guiming Yang)	0.396	3000	61353.3835
32	d4(Guiming Yang)	0.44	6000	83007.5188
33	E1(Jinpeng Zhou)	0.465	1500	15112.7820
34	E2(Jinpeng Zhou)	0.412	3000	54135.3383
35	E3(Jinpeng Zhou)	0.472	3000	52030.0751
36	E4(Jinpeng Zhou)	0.412	3000	54135.3383
37	e1(Jinpeng Zhou)	0.448	3000	37894.7368
38	e2(Jinpeng Zhou)	0.461	3000	32030.0752
39	e3(Jinpeng Zhou)	0.468	3000	28872.1805
40	e4(Jinpeng Zhou)	0.408	4500	58626.1107
41	F1(Ye Jia)	0.407	3000	56390.9774
42	F2(Ye Jia)	0.416	3000	52330.8271
43	F3(Ye Jia)	0.39	4500	96090.2256
44	F4(Ye Jia)	0.37	6000	146165.4135
45	f1(Ye Jia)）	0.472	3000	27067.6692
46	f2(Ye Jia)	0.453	3000	35639.0977
47	f3(Ye Jia)	0.433	3000	44661.6541
48	f4(Ye Jia)）	0.445	4500	58872.1805
49	G1(Hengji Li)	0.437	1500	21428.5714
50	G2(Hengji Li)	0.485	3000	21203.0075
51	G3(Hengji Li)	0.306	1500	50977.4436
52	G4(Hengji Li)	0.46	3000	83590.2256
53	g1(Hengji Li)	0.447	900	11503.7594
54	g2(Hengji Li)	0.454	1500	17593.9850
55	g3(Hengji Li)	0.461	3000	32030.0752
56	g4(Hengji Li)	0.464	3000	30676.6917
57	H1(Yinlu Sun)	0.47	1200	11187.9699
58	H2(Yinlu Sun)	0.432	3000	45112.7820
59	H3(Yinlu Sun)	0.427	3000	47368.4211
60	H4(Yinlu Sun)	0.429	4500	69699.2481

61	h1(Yinlu Sun)	0.465	3000	30225.5639
62	h2(Yinlu Sun)	0.466	3000	29774.4361
63	h3(Yinlu Sun)	0.465	3000	30225.5639
64	h4(Yinlu Sun)	0.403	3000	58195.4887
81	I1(Yongyan Sun)	0.482	3000	22556.3910
82	I2(Yongyan Sun)	0.464	3000	30676.6917
83	I3(Yongyan Sun)	0.403	3000	58195.4887
84	I4(Yongyan Sun)	0.456	4500	51428.5714
85	i1(Yongyan Sun)	0.436	3000	43308.2707
86	i2(Yongyan Sun)	0.467	3000	29323.3083
87	i3(Yongyan Sun)	0.474	3000	26165.4135
88	i4(Yongyan Sun)	0.472	3000	27067.6692
89	J1(BixiYu)	0.463	3000	31127.8195
90	J2(BixiYu)	0.432	3000	45112.7820
91	J3(BixiYu)	0.415	3000	52781.9549
92	J4(BixiYu)	0.425	4500	72406.0150
93	j1(BixiYu)	0.437	3000	42857.1429
94	j2(BixiYu)	0.425	3000	48270.6767
95	j3(BixiYu)	0.443	4500	60225.5639
96	j4(BixiYu)	0.383	3000	67218.0451
97	K1(Nianqiang Qu)	0.463	3000	31127.8195
98	K2(Nianqiang Qu)	0.457	3000	33834.5865
99	K3(Nianqiang Qu)	0.44	3000	83590.2256
100	K4(Nianqiang Qu)	0.473	24000	212932.3308
101	k1(Nianqiang Qu)	0.443	3000	40150.3759
102	k2(Nianqiang Qu)	0.425	3000	48270.6767
103	k3(Nianqiang Qu)	0.473	3000	26616.5414
104	k4(Nianqiang Qu)	0.455	3000	34736.8421
105	L1(Kaiming Zhu)	0.409	3000	55488.7218
106	L2(Kaiming Zhu)	0.464	4500	46015.0376
107	L3(Kaiming Zhu)	0.434	3000	44210.5263
108	L4(Kaiming Zhu)	0.405	3000	57293.2331
109	l1(Kaiming Zhu)	0.46	3000	32481.2030
110	l2(Kaiming Zhu)	0.464	3000	30676.6917
111	l3(Kaiming Zhu)	0.396	3000	61353.3835
112	l4(Kaiming Zhu)	0.401	3000	59097.7444
