# Supplementary material for: Effects of caffeinated beverage ingestion on salivary antimicrobial proteins responses to acute exercise in the heat
Source: Front Nutr. 2022 Nov 15;9:973003. doi: 10.3389/fnut.2022.973003 (PMC9705747; doi:10.3389/fnut.2022.973003)
Supplement: Supplementary file 1 [file Data_Sheet_1.ZIP › date/sLac.pdf]

1. Human Lactoferrin（LF）Elisa 测定试剂盒

standard concentration （μg/ml）	Determination of OD value	Absolute OD value
0	0. 0665	0
0. 3	0. 2174	0. 1509
0. 6	0. 3814	0. 3149
1. 2	0. 6549	0. 5884
2. 4	1. 178	1. 1115
4. 8	2. 0189	1. 9524

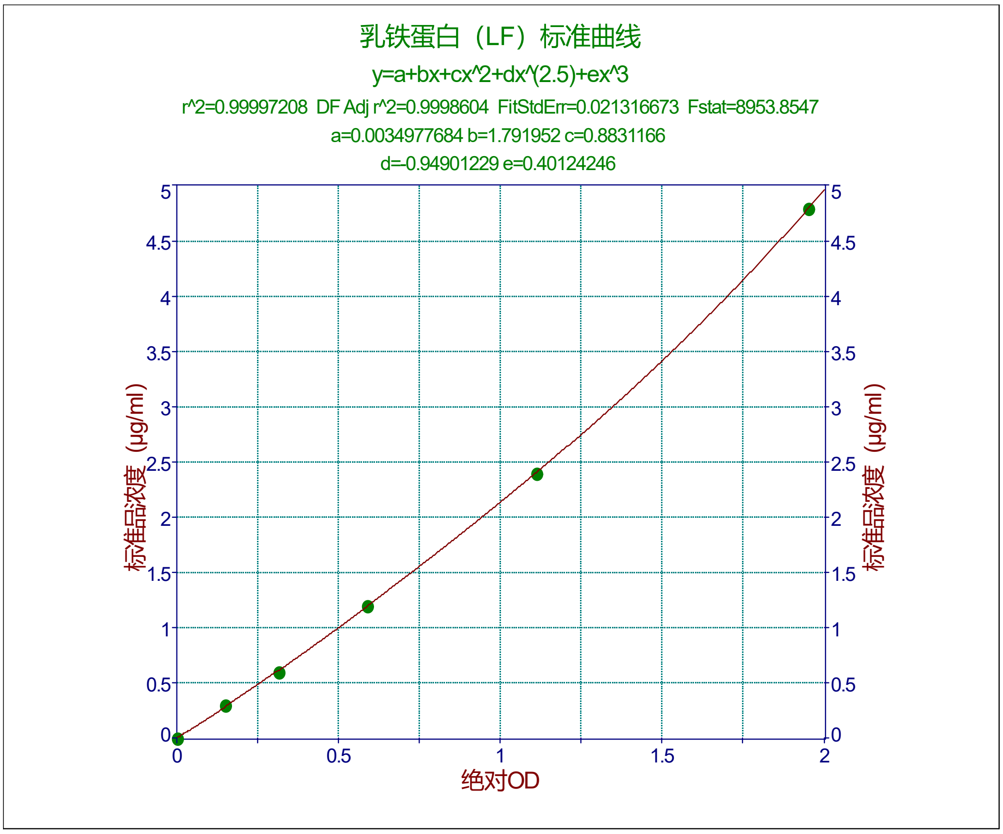

standard concentration （μg/ml）	Determination of OD value	Absolute OD value
0	0. 0598	0. 0000
0. 3	0. 2296	0. 1698
0. 6	0. 4104	0. 3506
1. 2	0. 7207	0. 6609
2. 4	1. 2849	1. 2251
4. 8	2. 0978	2. 0380

serial number	Sample number	OD	Saliva lactoferrin content（μg/ml）
1	A1(Zilun Zheng)	0.522	0.9080
2	A2(Zilun Zheng)	0.6125	1.1014
3	A3(Zilun Zheng)	0.5842	1.0405
4	A4(Zilun Zheng)	0.7441	1.3894
5	a1(Zilun Zheng)	0.497	0.8552
6	a2(Zilun Zheng)	0.4547	0.7666
7	a3(Zilun Zheng)	0.4688	0.7960
8	a4(Zilun Zheng)	0.7739	1.4557
9	B1(Qiang Bian)	0.7798	1.4688
10	B2(Qiang Bian)	0.9807	1.9280
11	B3(Qiang Bian)	1.4232	3.0275
12	B4(Qiang Bian)	1.57	3.4272
13	b1(Qiang Bian)	1.4755	1.1184
14	b2(Qiang Bian)	1.0463	2.0827
15	b3(Qiang Bian)	0.9295	1.8090
16	b4(Qiang Bian)	1.2692	2.6287
17	C1(Bowen Xing)	0.5573	0.9830
18	C2(Bowen Xing)	0.6097	1.0954
19	C3(Bowen Xing)	0.5399	1.2503
20	C4(Bowen Xing)	0.4784	1.4372
21	c1(Bowen Xing)	0.4687	0.7958
22	c2(Bowen Xing)	0.6152	1.1073
23	c3(Bowen Xing)	0.4994	0.8603
24	c4(Bowen Xing)	0.7716	1.4505
25	D1(Guiming Yang)	0.4001	0.6535
26	D2(Guiming Yang)	0.4521	0.7612
27	D3(Guiming Yang)	0.5697	1.0095
28	D4(Guiming Yang)	0.7075	1.3085
29	d1(Guiming Yang)	0.4131	0.6803
30	d2(Guiming Yang)	0.4293	0.7138
31	d3(Guiming Yang)	1.1663	2.3724
32	d4(Guiming Yang)	1.3758	2.9027

33	E1(Jinpeng Zhou)	0.6141	1.1049
34	E2(Jinpeng Zhou)	0.7605	1.4258
35	E3(Jinpeng Zhou)	0.7068	1.3069
36	E4(Jinpeng Zhou)	0.7153	1.3257
37	e1(Jinpeng Zhou)	0.5805	1.0326
38	e2(Jinpeng Zhou)	0.4727	0.8042
39	e3(Jinpeng Zhou)	1.0919	2.1917
40	e4(Jinpeng Zhou)	1.3299	2.7836
41	F1(Ye Jia)	0.6086	1.0930
42	F2(Ye Jia)	0.5806	1.0328
43	F3(Ye Jia)	0.7933	1.4991
44	F4(Ye Jia)	0.9494	1.8551
45	f1(Ye Jia)）	0.5517	0.9711
46	f2(Ye Jia)	0.5614	0.9918
47	f3(Ye Jia)	0.5404	0.9470
48	f4(Ye Jia)）	0.6541	1.1916
49	G1(Hengji Li)	0.4844	0.8287
50	G2(Hengji Li)	0.4364	0.7285
51	G3(Hengji Li)	0.5496	0.9666
52	G4(Hengji Li)	0.4656	1.4993
53	g1(Hengji Li)	0.4288	0.7127
54	g2(Hengji Li)	0.4514	0.7597
55	g3(Hengji Li)	0.48023	0.8200
56	g4(Hengji Li)	0.4707	0.8000
57	H1(Yinlu Sun)	0.4965	0.8542
58	H2(Yinlu Sun)	0.6476	1.1775
59	H3(Yinlu Sun)	0.5635	1.2756
60	H4(Yinlu Sun)	0.7418	1.3843
61	h1(Yinlu Sun)	0.4982	0.8577
62	h2(Yinlu Sun)	0.4707	0.8000
63	h3(Yinlu Sun)	0.496	0.8531
64	h4(Yinlu Sun)	0.743	1.3869
81	I1(Yongyan Sun)	0.4379	0.7316
82	I2(Yongyan Sun)	0.5483	0.9638
83	I3(Yongyan Sun)	0.5822	1.0363
84	I4(Yongyan Sun)	0.647	1.1762
85	i1(Yongyan Sun)	0.6423	1.1659
86	i2(Yongyan Sun)	0.5764	1.0238
87	i3(Yongyan Sun)	0.5275	0.9196

88	i4(Yongyan Sun)	0.5436	0.9538
89	J1(BixiYu)	0.4782	0.8157
90	J2(BixiYu)	0.5224	0.9088
91	J3(BixiYu)	0.5404	0.8510
92	J4(BixiYu)	0.5666	0.9001
93	j1(BixiYu)	0.5494	0.8678
94	j2(BixiYu)	0.5807	0.9266
95	j3(BixiYu)	0.6342	1.0280
96	j4(BixiYu)	0.7001	1.1547
97	K1(Nianqiang Qu)	0.551	0.8708
98	K2(Nianqiang Qu)	0.5784	0.9222
99	K3(Nianqiang Qu)	0.6767	1.1095
100	K4(Nianqiang Qu)	1.2106	2.2271
101	k1(Nianqiang Qu)	0.5511	0.8710
102	k2(Nianqiang Qu)	0.6155	0.9924
103	k3(Nianqiang Qu)	0.5095	0.7935
104	k4(Nianqiang Qu)	0.4933	0.7635
105	L1(Kaiming Zhu)	0.753	1.2580
106	L2(Kaiming Zhu)	0.7427	1.2377
107	L3(Kaiming Zhu)	0.7307	1.2143
108	L4(Kaiming Zhu)	0.7626	1.2769
109	l1(Kaiming Zhu)	0.8462	1.4436
110	l2(Kaiming Zhu)	0.7146	1.1829
111	l3(Kaiming Zhu)	0.7852	1.3216
112	l4(Kaiming Zhu)	0.8531	1.4576
