# Supplementary material for: Effects of caffeinated beverage ingestion on salivary antimicrobial proteins responses to acute exercise in the heat
Source: Front Nutr. 2022 Nov 15;9:973003. doi: 10.3389/fnut.2022.973003 (PMC9705747; doi:10.3389/fnut.2022.973003)
Supplement: Supplementary file 1 [file Data_Sheet_1.ZIP › sAA.docx]

| rial number | Sample number | OD | [dilution](javascript:;) [ratio](javascript:;) | [amylase](javascript:;) [activity](javascript:;)（U/dl） |
| --- | --- | --- | --- | --- |
|  | [standard](javascript:;) | 0.532 |  |  |
| 1 | A1(Zilun Zheng) | 0.405 | 3000 | 57293.2331 |
| 2 | A2(Zilun Zheng) | 0.436 | 4500 | 64962.4060 |
| 3 | A3(Zilun Zheng) | 0.399 | 3000 | 60000.0000 |
| 4 | A4(Zilun Zheng) | 0.41 | 6000 | 110075.1880 |
| 5 | a1(Zilun Zheng) | 0.442 | 3000 | 40601.5038 |
| 6 | a2(Zilun Zheng) | 0.456 | 3000 | 34285.7143 |
| 7 | a3(Zilun Zheng) | 0.438 | 3000 | 42406.0150 |
| 8 | a4(Zilun Zheng) | 0.429 | 4500 | 69699.2481 |
| 9 | B1(Qiang Bian) | 0.457 | 3000 | 33834.5865 |
| 10 | B2(Qiang Bian) | 0.421 | 3000 | 50075.1880 |
| 11 | B3(Qiang Bian) | 0.408 | 3000 | 55939.8496 |
| 12 | B4(Qiang Bian) | 0.399 | 4500 | 90000.0000 |
| 13 | b1(Qiang Bian) | 0.429 | 3000 | 46466.1654 |
| 14 | b2(Qiang Bian) | 0.411 | 3000 | 54586.4662 |
| 15 | b3(Qiang Bian) | 0.444 | 3000 | 39699.2481 |
| 16 | b4(Qiang Bian) | 0.425 | 4500 | 72406.0150 |
| 17 | C1(Bowen Xing) | 0.378 | 1500 | 34736.8421 |
| 18 | C2(Bowen Xing) | 0.389 | 2400 | 51609.0226 |
| 19 | C3(Bowen Xing) | 0.455 | 3000 | 34736.8421 |
| 20 | C4(Bowen Xing) | 0.437 | 3000 | 42857.1429 |
| 21 | c1(Bowen Xing) | 0.468 | 3000 | 28872.1805 |
| 22 | c2(Bowen Xing) | 0.47 | 3000 | 27969.9248 |
| 23 | c3(Bowen Xing) | 0.445 | 3000 | 39248.1203 |
| 24 | c4(Bowen Xing) | 0.377 | 3000 | 69924.8120 |
| 25 | D1(Guiming Yang) | 0.479 | 3000 | 23909.7744 |
| 26 | D2(Guiming Yang) | 0.473 | 3000 | 26616.5414 |
| 27 | D3(Guiming Yang) | 0.409 | 3000 | 55488.7218 |
| 28 | D4(Guiming Yang) | 0.438 | 4500 | 63609.0226 |

| 29 | d1(Guiming Yang) | 0.482 | 3000 | 22556.3910 |
| --- | --- | --- | --- | --- |
| 30 | d2(Guiming Yang) | 0.478 | 1500 | 12180.4511 |
| 31 | d3(Guiming Yang) | 0.396 | 3000 | 61353.3835 |
| 32 | d4(Guiming Yang) | 0.44 | 6000 | 83007.5188 |
| 33 | E1(Jinpeng Zhou) | 0.465 | 1500 | 15112.7820 |
| 34 | E2(Jinpeng Zhou) | 0.412 | 3000 | 54135.3383 |
| 35 | E3(Jinpeng Zhou) | 0.472 | 3000 | 52030.0751 |
| 36 | E4(Jinpeng Zhou) | 0.412 | 3000 | 54135.3383 |
| 37 | e1(Jinpeng Zhou) | 0.448 | 3000 | 37894.7368 |
| 38 | e2(Jinpeng Zhou) | 0.461 | 3000 | 32030.0752 |
| 39 | e3(Jinpeng Zhou) | 0.468 | 3000 | 28872.1805 |
| 40 | e4(Jinpeng Zhou) | 0.408 | 4500 | 58626.1107 |
| 41 | F1(Ye Jia) | 0.407 | 3000 | 56390.9774 |
| 42 | F2(Ye Jia) | 0.416 | 3000 | 52330.8271 |
| 43 | F3(Ye Jia) | 0.39 | 4500 | 96090.2256 |
| 44 | F4(Ye Jia) | 0.37 | 6000 | 146165.4135 |
| 45 | f1(Ye Jia)） | 0.472 | 3000 | 27067.6692 |
| 46 | f2(Ye Jia) | 0.453 | 3000 | 35639.0977 |
| 47 | f3(Ye Jia) | 0.433 | 3000 | 44661.6541 |
| 48 | f4(Ye Jia)） | 0.445 | 4500 | 58872.1805 |
| 49 | G1(Hengji Li) | 0.437 | 1500 | 21428.5714 |
| 50 | G2(Hengji Li) | 0.485 | 3000 | 21203.0075 |
| 51 | G3(Hengji Li) | 0.306 | 1500 | 50977.4436 |
| 52 | G4(Hengji Li) | 0.46 | 3000 | 83590.2256 |
| 53 | g1(Hengji Li) | 0.447 | 900 | 11503.7594 |
| 54 | g2(Hengji Li) | 0.454 | 1500 | 17593.9850 |
| 55 | g3(Hengji Li) | 0.461 | 3000 | 32030.0752 |
| 56 | g4(Hengji Li) | 0.464 | 3000 | 30676.6917 |
| 57 | H1(Yinlu Sun) | 0.47 | 1200 | 11187.9699 |
| 58 | H2(Yinlu Sun) | 0.432 | 3000 | 45112.7820 |
| 59 | H3(Yinlu Sun) | 0.427 | 3000 | 47368.4211 |
| 60 | H4(Yinlu Sun) | 0.429 | 4500 | 69699.2481 |

| 61 | h1(Yinlu Sun) | 0.465 | 3000 | 30225.5639 |
| --- | --- | --- | --- | --- |
| 62 | h2(Yinlu Sun) | 0.466 | 3000 | 29774.4361 |
| 63 | h3(Yinlu Sun) | 0.465 | 3000 | 30225.5639 |
| 64 | h4(Yinlu Sun) | 0.403 | 3000 | 58195.4887 |
| 81 | I1(Yongyan Sun) | 0.482 | 3000 | 22556.3910 |
| 82 | I2(Yongyan Sun) | 0.464 | 3000 | 30676.6917 |
| 83 | I3(Yongyan Sun) | 0.403 | 3000 | 58195.4887 |
| 84 | I4(Yongyan Sun) | 0.456 | 4500 | 51428.5714 |
| 85 | i1(Yongyan Sun) | 0.436 | 3000 | 43308.2707 |
| 86 | i2(Yongyan Sun) | 0.467 | 3000 | 29323.3083 |
| 87 | i3(Yongyan Sun) | 0.474 | 3000 | 26165.4135 |
| 88 | i4(Yongyan Sun) | 0.472 | 3000 | 27067.6692 |
| 89 | J1(BixiYu) | 0.463 | 3000 | 31127.8195 |
| 90 | J2(BixiYu) | 0.432 | 3000 | 45112.7820 |
| 91 | J3(BixiYu) | 0.415 | 3000 | 52781.9549 |
| 92 | J4(BixiYu) | 0.425 | 4500 | 72406.0150 |
| 93 | j1(BixiYu) | 0.437 | 3000 | 42857.1429 |
| 94 | j2(BixiYu) | 0.425 | 3000 | 48270.6767 |
| 95 | j3(BixiYu) | 0.443 | 4500 | 60225.5639 |
| 96 | j4(BixiYu) | 0.383 | 3000 | 67218.0451 |
| 97 | K1(Nianqiang Qu) | 0.463 | 3000 | 31127.8195 |
| 98 | K2(Nianqiang Qu) | 0.457 | 3000 | 33834.5865 |
| 99 | K3(Nianqiang Qu) | 0.44 | 3000 | 83590.2256 |
| 100 | K4(Nianqiang Qu) | 0.473 | 24000 | 212932.3308 |
| 101 | k1(Nianqiang Qu) | 0.443 | 3000 | 40150.3759 |
| 102 | k2(Nianqiang Qu) | 0.425 | 3000 | 48270.6767 |
| 103 | k3(Nianqiang Qu) | 0.473 | 3000 | 26616.5414 |
| 104 | k4(Nianqiang Qu) | 0.455 | 3000 | 34736.8421 |
| 105 | L1(Kaiming Zhu) | 0.409 | 3000 | 55488.7218 |
| 106 | L2(Kaiming Zhu) | 0.464 | 4500 | 46015.0376 |
| 107 | L3(Kaiming Zhu) | 0.434 | 3000 | 44210.5263 |
| 108 | L4(Kaiming Zhu) | 0.405 | 3000 | 57293.2331 |
| 109 | l1(Kaiming Zhu) | 0.46 | 3000 | 32481.2030 |
| 110 | l2(Kaiming Zhu) | 0.464 | 3000 | 30676.6917 |
| 111 | l3(Kaiming Zhu) | 0.396 | 3000 | 61353.3835 |
| 112 | l4(Kaiming Zhu) | 0.401 | 3000 | 59097.7444 |
