# Supplementary material for: Effects of caffeinated beverage ingestion on salivary antimicrobial proteins responses to acute exercise in the heat
Source: Front Nutr. 2022 Nov 15;9:973003. doi: 10.3389/fnut.2022.973003 (PMC9705747; doi:10.3389/fnut.2022.973003)
Supplement: Supplementary file 2 [file Data_Sheet_2.docx]

Supplementary Material

# Supplementary Figures and Tables

## Supplementary Figures


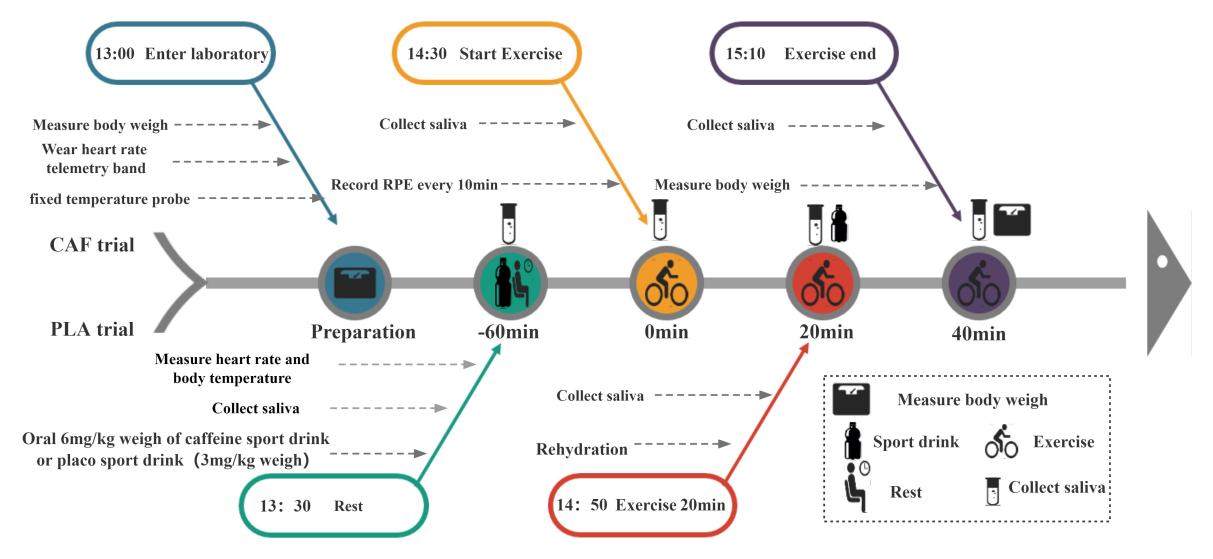


Figure 1. Experimental procedure chart

Table 1. Changes in sAA and sLac over the entire trial (‾*x* ± *s,* *n*=12)

| Measurements | Conditions | T_-60_ | T_0_ | T_20_ | T_40_ |
| --- | --- | --- | --- | --- | --- |
| sLac (μg/ml) | CAF | 0.96±0.23 | 1.11±0.32 | 1.30±0.57^*^ | 1.60±0.66^**#△^ |
|  | CON | 0.95±0.21 | 1.01±0.37 | 1.23±0.57 | 1.58±0.76^**#△^ |
| s AA (U/L) | CAF | 3285±1588 | 4347±1284^**^ | 5203±1702^**#▲^ | 7281±1769^**#△^ |
|  | CON | 3367±1020 | 3338±1231 | 4107±1345 | 5746±1769^**#△^ |

^*^*P*<0.05，^**^*P*<0.01 *vs* T_-60_；^#^ *P*<0.01 *vs* T_0_；^△^ *P*<0.01 *vs* T_20_；^▲^*P*<0.05 *vs* PLA；


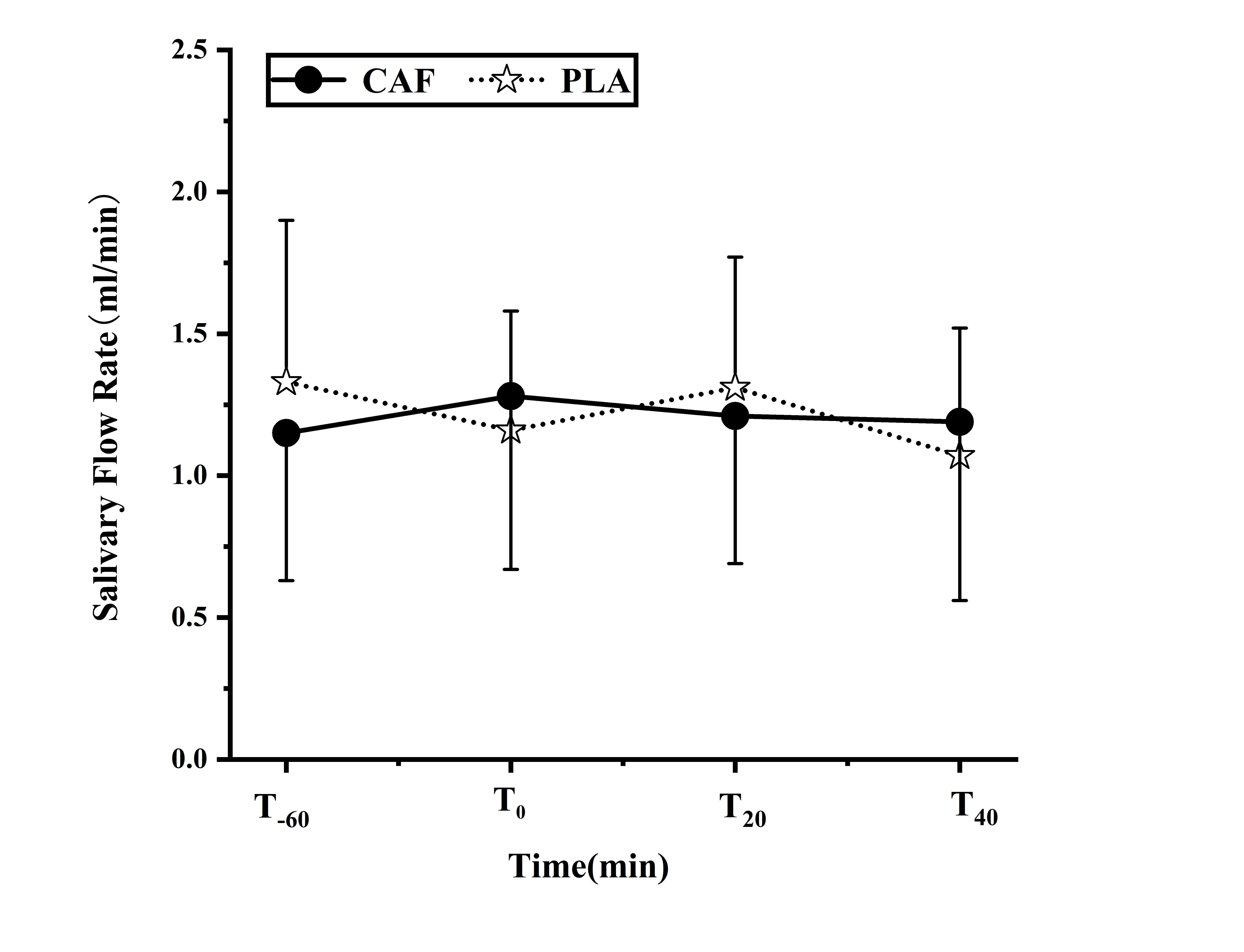


Figure.2 Saliva flow rate during the two trials of the participants


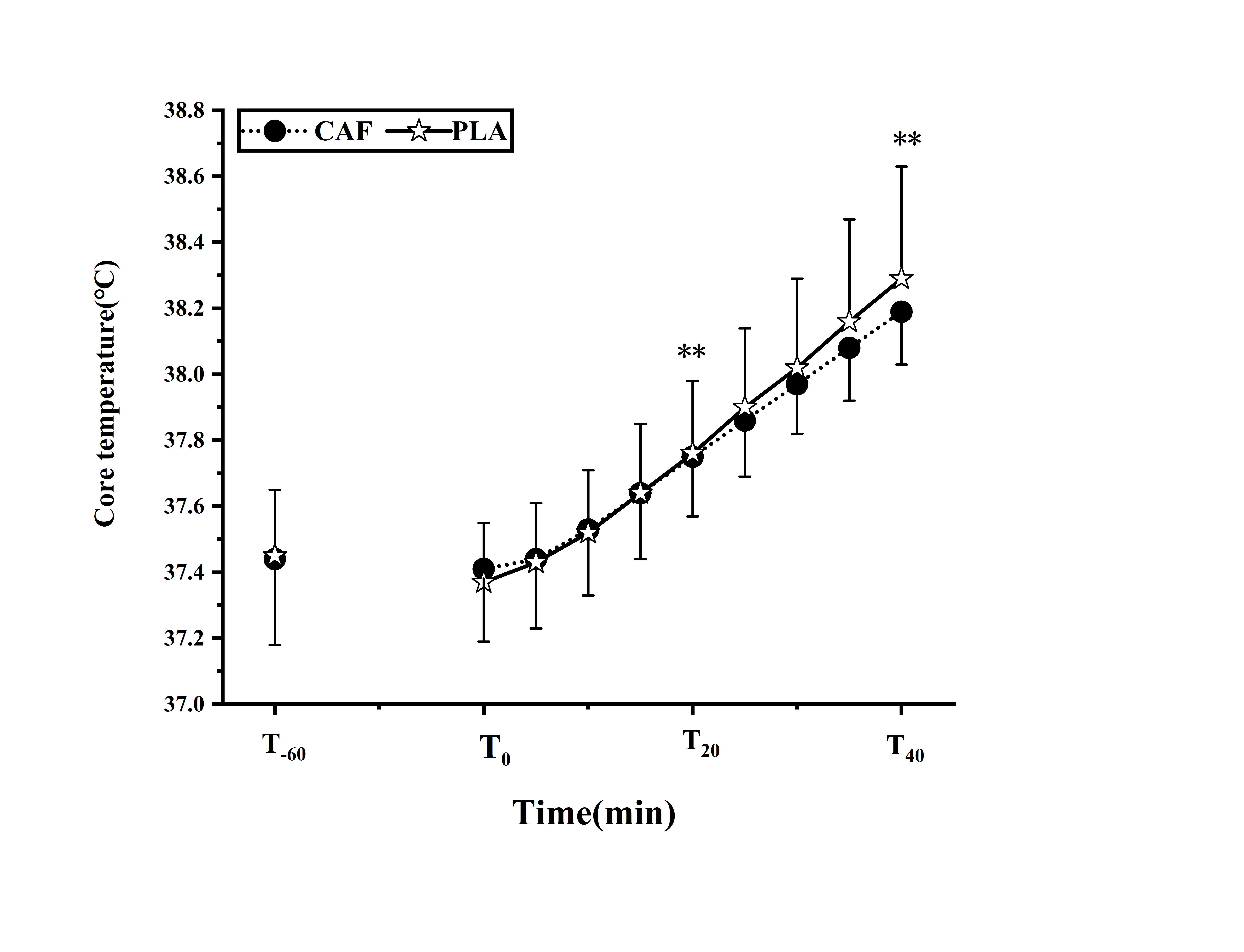


Figure 3. The change of core temperature in two trials.

^**^*P*<0.01 *vs* T_0_；

| Conditions | Pre-exercise weight (kg) | Post-exercise weight (kg) | Sweat volume (kg) | Weight Loss (%) |
| --- | --- | --- | --- | --- |
| CAF | 72.03±6.84 | 71.63±6.80^**^ | 0.40±0.12 | 0.55±0.18 |
| PLA | 72.13±6.67 | 71.78±6.63^**^ | 0.35±0.12 | 0.48±0.14 |

Table 2. Changes in sweat volume and weight loss of the participants during trials (‾*x* ± *s,* *n*=12)

^**^*P*<0.01 vs pre-exercise weight

Table 3. Heart rate responses over the entire trial (beats/min*，*‾*x* ± *s,* *n*=12)

| Measurements | Conditions | T_-60_ | T_0_ | T_20_ | T_40_ |
| --- | --- | --- | --- | --- | --- |
| HR | CAF | 81.08±14.06 | 76.17±13.52 | 139.92±13.22^**^ | 153.67±15.14^**#^ |
|  | PLA | 80.17±14.75 | 83.25±12.73 | 142.50±14.86^**^ | 154.42±15.71^**#^ |

^**^*P*<0.01 *vs* T_0_；^#^ *P*<0.01 *vs* T_20_；

Table 4. Changes in RPE during exercise in both trials (‾*x* ± *s,* *n*=12)

| Measurements | Conditions | T_0_ | T_10_ | T_20_ | T_30_ | T_40_ |
| --- | --- | --- | --- | --- | --- | --- |
| RPE | CAF | 8.75±1.60 | 9.42±1.78 | 10.17±1.95^**##^ | 11.00±2.17^**##△△○○^ | 12.25±2.86^**##△△▲▲○^ |
|  | CON | 8.67±2.10 | 10.58±2.39^**^ | 11.75±1.87^**^ | 13.42±1.83^**##△△^ | 14.83±2.79^**##△△▲▲^ |

***P*<0.01 *vs* T_0_；^##^*P*<0.01 *vs* T_10_； ^△△^*P*<0.01 *vs* T_20_；^▲▲^*P*<0.01 *vs* T_30_；^○^*P*<0.05,^○○^*P*<0.01 *vs* PLA；
